# Muscle-Invasive Bladder Paraganglioma: Case Report and Comprehensive Literature Review

**DOI:** 10.7759/cureus.95046

**Published:** 2025-10-21

**Authors:** Rodrigo Escalante-Armenta, Juan Blanco-Gonzalez, Cristian-Axel Hernandez-Gaytan, Horst Emanuel Lagos Beitz, Guillermo Trujillo-Martinez, Guillermo H Martínez-Delgado, Brenda Bautista Martinez, Julian Arista, Ricardo A Castillejos-Molina

**Affiliations:** 1 Urology, Instituto Nacional de Ciencias Médicas y Nutrición "Salvador Zubirán", Mexico City, MEX; 2 Pathology, Instituto Nacional de Ciencias Médicas y Nutrición "Salvador Zubirán", Mexico City, MEX

**Keywords:** bladder paraganglioma, case report, immunohistochemistry, muscle invasive tumor, neuroendocrine tumor, rare bladder tumor, urinary bladder neoplasm, urologic oncology

## Abstract

Bladder paragangliomas are rare neuroendocrine tumors that represent a minimal fraction of bladder neoplasms and often pose diagnostic challenges due to nonspecific symptoms. Arising from chromaffin tissue within the bladder wall, they may present with hematuria, paroxysmal hypertension, headache, or palpitations, frequently leading to misdiagnosis as other bladder tumors. We report the case of a 72-year-old man with hypertension admitted for gross hematuria, headaches, and paroxysms. Imaging revealed a 5.4×4.4 cm hypervascular mass on the right posterolateral bladder wall, confirmed cystoscopically. Pathology from transurethral resection showed a muscle-invasive paraganglioma with characteristic immunohistochemistry. Biochemical testing demonstrated elevated catecholamine metabolites, and staging excluded metastasis. The patient underwent open cystoprostatectomy with Bricker-type ileal conduit; final pathology confirmed a large paraganglioma without adverse features. Postoperatively, he remains asymptomatic and normotensive without medication. This case highlights the need to consider bladder paraganglioma in patients with hematuria and hypertensive episodes. Accurate diagnosis relies on biochemical, imaging, and histopathologic correlation, and complete surgical excision remains the cornerstone of management through a multidisciplinary approach.

## Introduction

Paraganglioma is an unusual neoplasm originating from extra-adrenal chromaffin cells, specifically within the paravertebral ganglia of the sympathetic nervous system. While 80%-85% of chromaffin cell tumors are pheochromocytomas, the remaining 15%-20% are classified as paragangliomas [1].

In terms of location, extra-adrenal paragangliomas are more commonly found in non-genitourinary sites (93%), with genitourinary locations being less frequent (6.7%) [2]. Within the genitourinary system, the bladder is the predominant site for these neoplasms, accounting for approximately 72.2% of cases [3]. However, bladder paragangliomas are extremely rare, representing only 0.06% of all bladder tumors [4]. To date, approximately 200 cases have been documented worldwide [5].

These tumors arise from chromaffin tissue in the sympathetic nervous system and are embedded in the muscular layer of the bladder wall. Bladder paragangliomas can occur across all age groups but are most commonly diagnosed in individuals between 20 and 50 years of age, with a slight female predominance [5]. Clinically, bladder paragangliomas may be incidentally detected during routine physical examinations or may present with symptoms such as paroxysmal hypertension, hematuria, and, in some cases, episodes of headache or palpitations [6]. The nonspecific nature of these symptoms, combined with their rarity, often leads to delayed or incorrect diagnosis, complicating clinical management. Identifying functional bladder paragangliomas before surgery is crucial, as catecholamine-secreting tumors may trigger severe hypertensive crises during manipulation. Recognizing this possibility preoperatively allows for adequate medical preparation and safer intraoperative management.

Surgical resection is the most effective treatment for bladder paragangliomas. According to a systematic review of the literature, partial cystectomy is the most commonly performed surgical approach, accounting for 68.9% of cases. Other options for localized tumors include transurethral resection of bladder tumors (TURBT, 19.8%) and, less frequently, radical cystectomy (11.3%) [5].

The surgical management of this entity remains challenging due to its rarity and the variability in technical preferences. These findings underscore the importance of a multidisciplinary approach to optimize the diagnosis, treatment, and follow-up of patients with bladder paragangliomas.

## Case presentation

A 72-year-old man with a past medical history significant for hypertension treated with amlodipine and losartan was referred to our hospital with the antecedent of gross hematuria, headache and paroxysms. The patient denied a relevant family history.

The vital signs in the admission were: heart rate 105 beats per minute with a blood pressure of 158/88 mmHg, the remaining vital signs were within normal limits.

**Figure 1:**
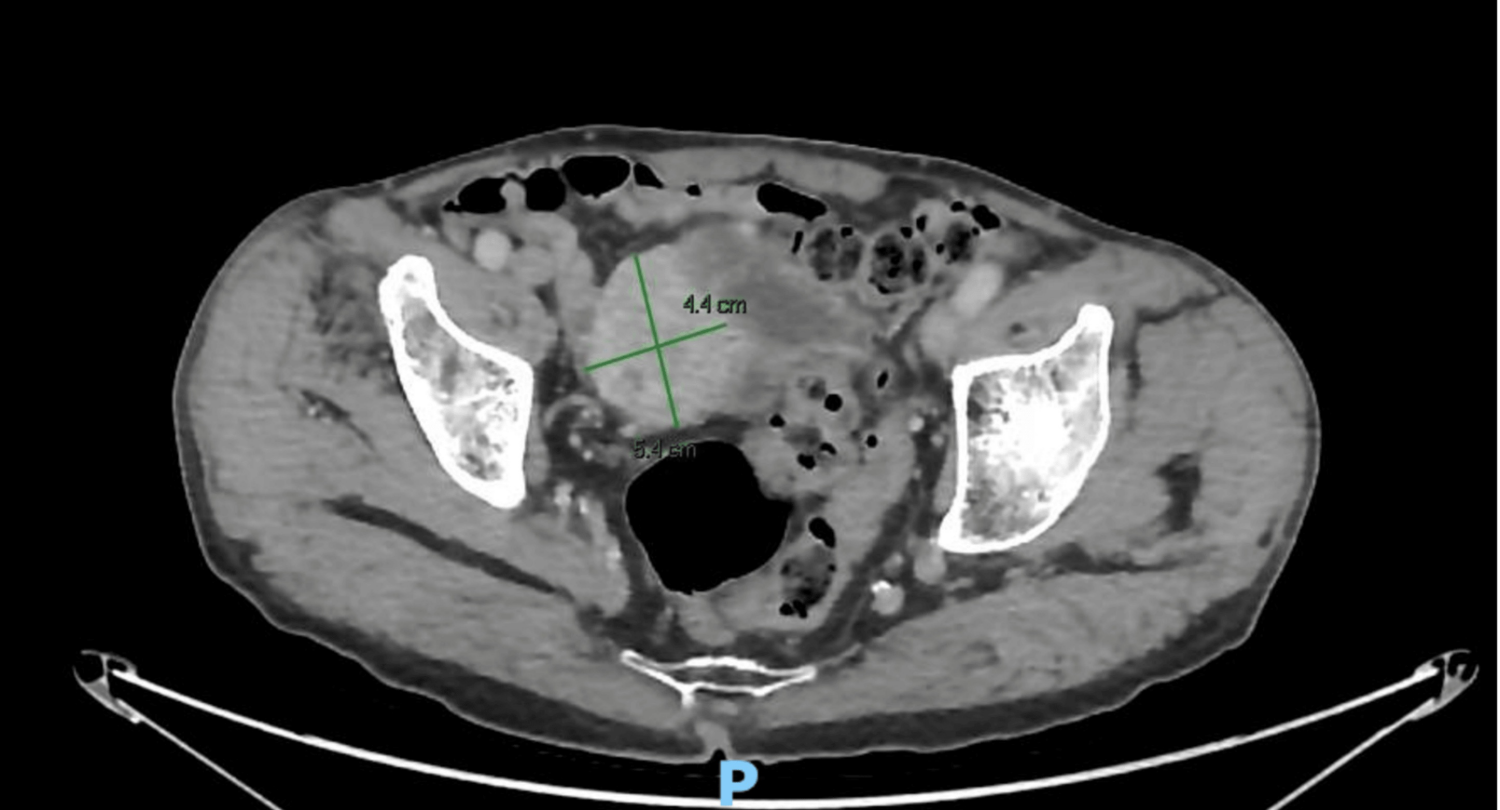
Contrast-enhanced CT imaging with a solid lesion of 5.4×4.4 cm on the right posterolateral wall of the bladder, with irregular margins

Clinically, the patient appeared calm and hemodynamically stable, on physical examination, no pelvic mass was detected. A CT urography was done and revealed a solid lesion on the right posterolateral wall of the bladder with irregular edges and arterial enhancement, with exophytic growth and peripheral calcifications, without invading adjacent structures. The mass measured 5.4x4.3 cm (Figure 1).

Cystoscopy revealed a 4 cm solid tumor, as shown in the right posterolateral wall of the bladder. A TURB was done, which reported a muscle-invasive bladder paraganglioma. Immunohistochemical analysis showed positive expression for S100, GATA3, and Ki-67 (2%), while succinate dehydrogenase (SDH) was negative.

We performed the urine metanephrine test as part of laboratory studies, in which normetanephrine was 4091 µg/24 hours, metanephrine 39 µg/24 hours, plasmatic metanephrine 110 pg/mL and normetanephrine >2400 pg/mL (Table 1).

**Table 1:** Laboratory results with reference values. Abbreviations: µg=microgram, pg=picogram, mL=milliliter.

Test	Result	Reference value
Urine normetanephrine (24 h)	4091 µg/24h	110-695 µg/24h
Urine metanephrine (24 h)	39 µg/24h	24-96 µg/24h
Plasma metanephrine	110 pg/mL	<90 pg/mL
Plasma normetanephrine	>2400 pg/mL	<180 pg/mL

To complement the diagnostic approach, a thoracic CT scan and a PET DOTA (1,4,7,10-tetraazacyclododecane-1,4,7,10-tetraacetic acid) were performed, in which no metastases were observed.

**Figure 2:**
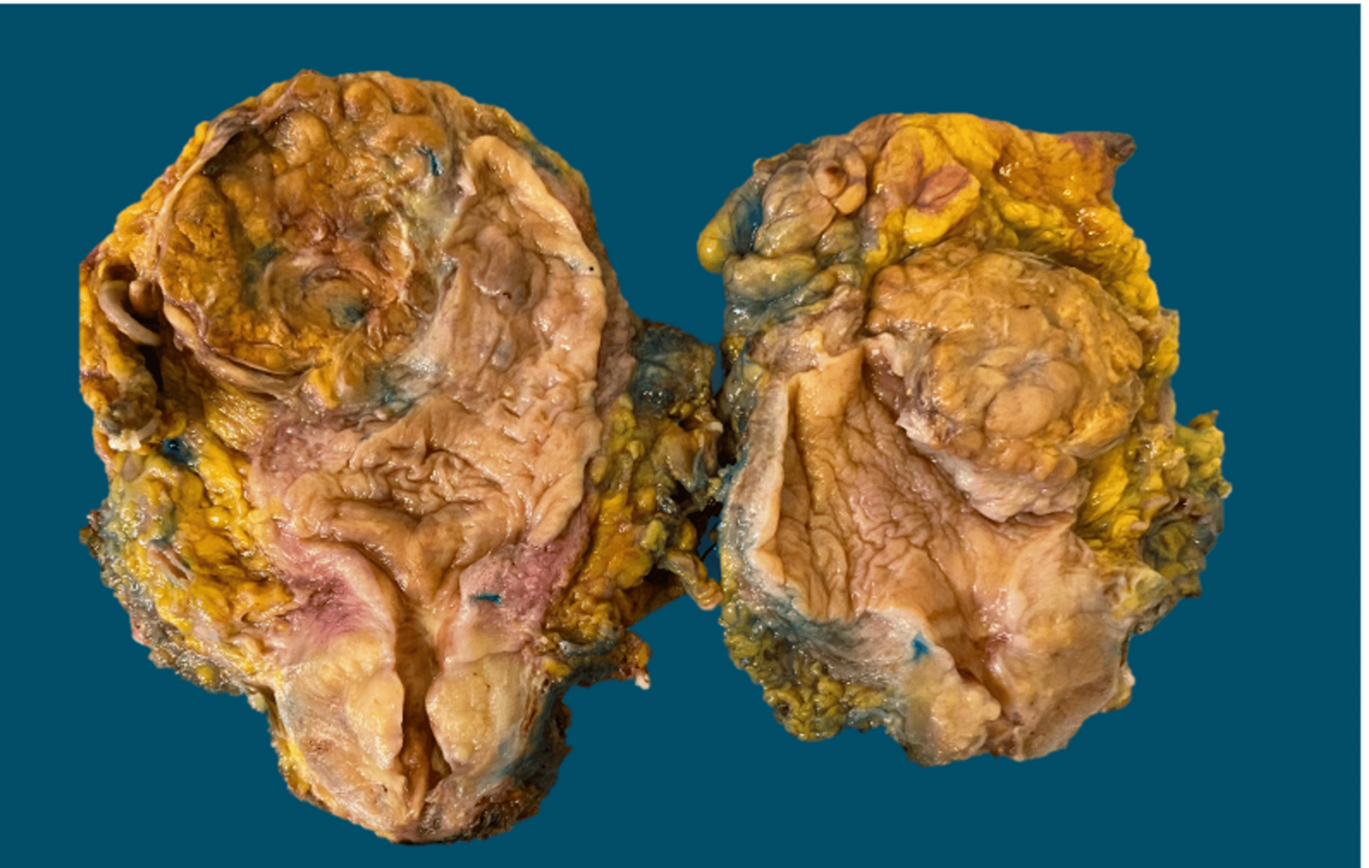
Bladder gross specimen. Tumor located on the right lateral wall

An open cystoprostatectomy, and Bricker-type ileal conduit were performed, revealing a 5 cm hypervascular bladder tumor. Ureteral margins were negative intraoperatively. The surgical specimen revealed a bladder with dimensions 90x80 mm and prostate 50x40x35 mm; the lesion size was 70x45x45 mm (Figure 2).

**Figure 3:**
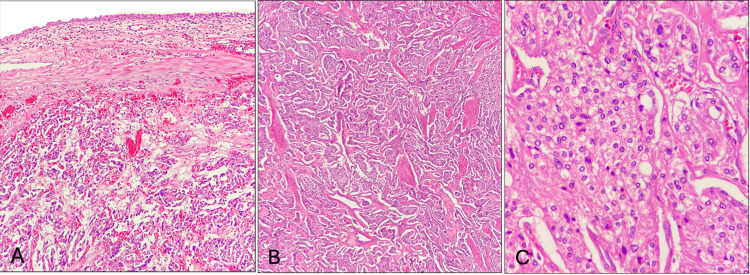
Histologic features of bladder paraganglioma. (A) Neoplastic cells infiltrating the detrusor (hematoxylin and eosin (H&E) stain, 40x). (B) Zellballen pattern with fibrovascular septa (H&E, 100x). (C) Tumor cells with eosinophilic granular cytoplasm and monomorphic nuclei without necrosis or atypia (H&E, 400x).

Histopathology demonstrated tumor cells beneath the urothelium infiltrating the bladder muscle in a zellballen pattern. Cytoplasm was eosinophilic and granular with monomorphic nuclei and no necrosis or vascular invasion (Figure 3).

The patient remains asymptomatic on outpatient oncological follow-up, with adequate blood pressure levels, without the use of antihypertensive drugs, and negative metanephrines.

## Discussion

Diagnostic modalities

Biochemical evaluation plays a pivotal role in confirming suspected bladder paragangliomas. Plasma-free metanephrines are the recommended initial screening tool due to their high sensitivity (97%) and specificity (96%), outperforming catecholamine measurements [6]. This superiority is attributed to the activity of catechol-O-methyltransferase (COMT), which converts catecholamines into more stable metanephrines.

As a confirmatory test, 24-hour urine catecholamines and metanephrines are highly specific (96%) and complement initial plasma tests despite slightly lower sensitivity (79%) [6]. However, intermittent catecholamine secretion in these tumors often results in a low positivity rate (~60%) [7,8]. To improve diagnostic accuracy, measurements of plasma catecholamines before, during, and after micturition are recommended, particularly in patients with micturition attacks [9,10].

Imaging is equally critical in characterizing the anatomical and functional features of bladder paragangliomas and aiding in differential diagnosis. Ultrasound is a useful initial modality, where tumors typically appear as well-demarcated hypoechoic lesions with increased vascularity on color Doppler imaging [1, 5]. Computed tomography (CT) remains the gold standard for evaluating bladder masses, especially in patients with gross hematuria. Non-contrast CT scans reveal homogeneous, soft-tissue density lesions (19.9-52 Hounsfield units (HU)), while contrast-enhanced scans demonstrate significant arterial phase enhancement (64.3-117.9 HU), indicative of their hypervascular nature [3,11,12].

Magnetic resonance imaging (MRI) provides superior soft-tissue contrast and is particularly valuable for identifying tumors in deeper layers of the bladder wall. Bladder paragangliomas often appear hyperintense on T1- and T2-weighted images, with larger lesions displaying the characteristic “salt-and-pepper” pattern due to their high vascularity [12]. Functional imaging modalities such as PET-CT with gallium-68 DOTA-(Tyr3)-octreotate (DOTATATE) or ^131^I-MIBG (metaiodobenzylguanidine) scintigraphy are also helpful for detecting metastatic or multifocal disease. These techniques offer sensitivity rates of 80%-95%, although their application may be limited by cost and equipment availability [13-15].

Staging

According to the National Comprehensive Cancer Network (NCCN) guidelines, the paraganglioma is classified by the TNM, T (Primary Tumor) classification defines the size and extent of the primary tumor in pheochromocytoma and sympathetic paraganglioma. If the tumor cannot be assessed, it is categorized as TX. T1 includes tumors smaller than 5 cm without extra-adrenal invasion, while T2 consists of tumors 5 cm or larger or any sympathetic paraganglioma, provided there is no invasion beyond the adrenal gland. T3 represents tumors of any size that have spread to surrounding tissues such as the liver, pancreas, spleen, or kidneys, while N0 indicates no regional lymph node metastasis. If metastasis to regional lymph nodes is present, it is classified as N1, and M0 indicates no distant metastasis, meaning the cancer remains localized. M1, on the other hand, signifies the presence of distant metastases, showing that the disease has spread to other parts of the body. M1 is further divided into three subcategories based on the metastatic locations: M1a, where metastasis is limited only to bone; M1b, where metastasis occurs only in distant lymph nodes, liver, or lung; and M1c, which represents the most advanced stage, with metastases in bone plus multiple other distant sites [16,17].

Surgical management and therapeutic considerations

Surgical resection remains the mainstay of treatment for bladder paragangliomas. Options include transurethral resection (TUR), partial cystectomy, or radical cystectomy for advanced cases. While TUR is minimally invasive and technically straightforward, it poses significant challenges in functional tumors due to the potential for excessive catecholamine release during manipulation, leading to severe hypertensive crises. Additionally, the deep intramural location of these tumors often complicates complete resection, increasing the risk of recurrence [14,15].

Partial cystectomy is generally favored for most cases as it allows for more complete tumor removal while minimizing intraoperative risks. In a series of three patients treated with transurethral resection, two experienced significant hypertensive episodes during surgery, although these were successfully managed. This highlights the inherent risks of transurethral resection in functional tumors. Conversely, partial cystectomy has demonstrated superior oncologic outcomes, with a recurrence rate of approximately 15% compared to other techniques [15].

Preoperative preparation is critical to ensuring surgical safety. This involves the use of alpha-blockers, followed by beta-blockers, along with adequate hydration to mitigate the risk of hypertensive crises, arrhythmias, and strokes during the procedure [7]. Recent studies report that approximately 70% of patients with localized bladder paragangliomas have been successfully treated with partial cystectomy, reinforcing its role as the preferred therapeutic approach [5].

**Table 2:** Summary of Diagnostic, Staging, and Management Approaches in Bladder Paraganglioma MIBG: Metaiodobenzylguanidine; HU: Hounsfield units; DOTATATE: 1,4,7,10-Tetraazacyclododecane-1,4,7,10-tetraacetic acid-(Tyr3)-octreotate; NCCN: National Comprehensive Cancer Network; TNM: Tumor, Node, Metastasis.

Domain	Key Findings / Evidence	Diagnostic or Clinical Utility	References
Biochemical Evaluation	Plasma-free metanephrines are the preferred initial screening tool, superior to catecholamines due to COMT-mediated stability	First-line test for suspected cases of bladder paraganglioma	[6]
	24-hour urinary catecholamines and metanephrines confirm the diagnosis; intermittent secretion may limit positivity	Complements plasma tests to confirm biochemical diagnosis	[6–8]
	Measuring plasma catecholamines before, during, and after micturition improves diagnostic yield in patients with micturition attacks	Enhances detection of functional tumors	[9.10]
Imaging (Anatomical)	Ultrasound shows well-defined, hypoechoic, hypervascular lesions	Useful as an initial evaluation tool	[1,5]
	CT demonstrates homogeneous soft-tissue lesions (19.9–52 HU) with strong arterial enhancement (64.3–117.9 HU)	Gold standard for assessing bladder masses and local staging	[3,11,12]
	MRI shows hyperintense T1/T2 signals and “salt-and-pepper” appearance in large lesions	Superior soft-tissue delineation and depth assessment	[12]
Imaging (Functional)	PET-CT with Ga-68 DOTATATE and ^131^I-MIBG scintigraphy help identify multifocal or metastatic disease	Key for staging and detection of recurrence or distant spread	[13-15]
Staging (NCCN TNM)	T1<5 cm, no invasion; T2≥5 cm or sympathetic origin; T3=local organ invasion. N0/N1 for nodes; M0 localized; M1a-c distant metastases	Defines extent and prognosis according to NCCN criteria	[16,17]
Surgical Management	TUR allows minimal invasiveness but poses a risk of catecholamine crisis and incomplete resection	Reserved for diagnostic purposes or small, non-functional tumors	[14,15]
	Partial cystectomy enables complete tumor removal with lower recurrence (~15%)	Preferred surgical approach for localized tumors	[5,15]
	Radical cystectomy indicated for large or invasive tumors	Preferred surgical approach for localized tumors	[15]
Preoperative Preparation	α-Blockers followed by β-blockers and adequate hydration reduce perioperative risks	Essential to prevent hypertensive crises during surgery	[7]

## Conclusions

Bladder paraganglioma is an exceptionally rare entity that requires a multidisciplinary approach for accurate diagnosis and effective management. A combination of sensitive biochemical tests, advanced imaging techniques, and appropriately planned surgical interventions is essential to optimize patient outcomes. Partial cystectomy emerges as the treatment of choice for most cases, offering a balance of safety and long-term efficacy, particularly in functional or deeply located tumors.

Muscle-invasive bladder paraganglioma represents an uncommon manifestation of pheochromocytomas and paragangliomas. A high index of suspicion is warranted in patients presenting with symptoms such as headache, paroxysms, and hematuria. The management of this condition should involve an interdisciplinary team including endocrinologists, urologists, and anesthesiologists, with the support of specialists such as radiologists.
