# Correction: Muscle-Invasive Bladder Paraganglioma: Case Report and Comprehensive Literature Review

**DOI:** 10.7759/cureus.c376

**Published:** 2025-11-13

**Authors:** Rodrigo Escalante-Armenta, Juan Blanco-Gonzalez, Cristian-Axel Hernandez-Gaytan, Horst Emanuel Lagos Beitz, Guillermo Trujillo-Martinez, Guillermo H Martínez-Delgado, Brenda Bautista Martinez, Julian Arista, Ricardo A Castillejos-Molina

**Affiliations:** 1 Urology, Instituto Nacional de Ciencias Médicas y Nutrición "Salvador Zubirán", Mexico City, MEX; 2 Pathology, Instituto Nacional de Ciencias Médicas y Nutrición "Salvador Zubirán", Mexico City, MEX

This article has been corrected to revise the following measurement errors: 

- Abstract: "Imaging revealed a 5.4×**4.3** cm hypervascular..." corrected to "Imaging revealed a 5.4×**4.4** cm hypervascular..."
- Figure 1 Legend: "Contrast-enhanced CT imaging with a solid lesion of 5.4×**4.3** cm on the right..." corrected to "Contrast-enhanced CT imaging with a solid lesion of 5.4×**4.4** cm on the right..."

